# Synthesis and Spectroscopic Identification of Certain Imidazole-Semicarbazone Conjugates Bearing Benzodioxole Moieties: New Antifungal Agents

**DOI:** 10.3390/molecules24010200

**Published:** 2019-01-07

**Authors:** Reem I. Al-Wabli, Alwah R. Al-Ghamdi, Hazem A. Ghabbour, Mohamed H. Al-Agamy, Mohamed I. Attia

**Affiliations:** 1 Department of Pharmaceutical Chemistry, College of Pharmacy, King Saud University, P.O. Box 2457, Riyadh 11451, Saudi Arabia; toto24se@hotmail.com; 2 Department of Medicinal Chemistry, Faculty of Pharmacy, Mansoura University, Mansoura 35516, Egypt; ghabbourh@yahoo.com; 3 Department of Pharmaceutics, College of Pharmacy, King Saud University, P.O. Box 2457, Riyadh 11451, Saudi Arabia; malagamy@ksu.edu.sa; 4 Microbiology and Immunology Department, Faculty of Pharmacy, Al-Azhar University, Cairo 11884, Egypt; 5 Medicinal and Pharmaceutical Chemistry Department, Pharmaceutical and Drug Industries Research Division, National Research Centre (ID: 60014618), El Bohooth Street, Dokki, Giza 12622, Egypt

**Keywords:** crystal structure, imidazole, benzodioxole, semicarbazones, antifungal

## Abstract

During the last three decades the extent of life-threatening fungal infections has increased remarkably worldwide. Synthesis and structure elucidation of certain imidazole-semicarbazone conjugates **5a**–**o** are reported. Single crystal X-ray analysis of compound **5e** unequivocally confirmed its assigned chemical structure and the (*E*)-configuration of its imine double bond. Compound **5e** crystallized in the triclinic system, P-1, a = 6.3561 (3) Å, b = 12.5095 (8) Å, c = 14.5411 (9) Å, α = 67.073 (4)°, β = 79.989 (4)°, γ =84.370 (4)°, V = 1048.05 (11) Å^3^, Z = 2. In addition, DIZ and MIC assays were used to examine the in vitro antifungal activity of the title conjugates **5a**–**o** against four fungal strains. Compound **5e**, bearing a 4-ethoxyphenyl fragment, showed the best MIC value (0.304 µmol/mL) against both *C. tropicalis* and *C. parapsilosis* species, while compounds **5c** (MIC = 0.311 µmol/mL), **5k**, and **5l** (MIC = 0.287 µmol/mL) exhibited the best anti-*C. albicans* activity.

## 1. Introduction

The extent of life-threatening fungal infections has increased remarkably during the last three decades. Drug-resistance to the available antifungal agents and increasing morbidity due to life-threatening fungal infections have become a global health burden, particularly among individuals with organ transplants, AIDS, autoimmune diseases or those receiving anticancer chemotherapeutic agents [1,2,3,4]. Therefore, there is an urgent therapeutic need to develop new broad spectrum safe antifungal candidates with excellent activity toward various pathogenic fungi.

Azoles are the mainstay of antifungal therapy against different invasive fungal species owing to their broad antifungal spectrum and good pharmacokinetic profile [5]. Imidazole and 1,3,4-triazole moieties are the most common azole fragments in the therapeutically used antifungal azoles [6]. Azoles inhibit the fungal biosynthesis of ergosterol through inhibiting lanosterol 14α-demethylase (CYP51) which leads to accumulation of lanosterol and depletion of ergosterol along with the inability for normal growth of the fungi [7]. A two-carbon bridge separating the imidazole pharmacophore moiety and the aromatic portion of the molecule is prevalent in the available antifungal agents, while a three-carbon bridge is present in few antifungal candidates [8,9,10]. Thus, it was of interest to focus on the design of new imidazole-bearing compounds having a three-carbon bridge between the imidazole fragment and the aromatic moiety to examine their antifungal potential.

Semicarbazones are the products of an addition–elimination reaction between aldehydes or ketones with certain semicarbazides. In the solid state, semicarbazones principally exist in the keto-like form, while they manifest a keto-enol like tautomerism in the solution state giving rise to an effective electron delocalization along the semicarbazone fragment [11]. The presence of nitrogen and oxygen atoms as donor atoms in the core structure of semicarbazones gave them a special importance in both organic and medicinal chemistry due to their ability to coordinate with many metal ions [12]. Therefore, the research has recently focused on the biological importance of this type of compounds. An extensive literature review revealed that semicarbazones have exhibited a prominent antibacterial, anticonvulsant, antitumor, antioxidant, and antifungal activities [13,14,15,16]. 

The abovementioned premises persuaded us to design a series of imidazole-semicarbazone conjugates bearing 1,3-benzodioxole fragments. The incorporation of 1,3-benzodioxole moiety as an aromatic pharmacophore part in the title compounds **5a**–**o** could potentiate their antifungal potential [17,18]. Compounds **5a**–**o** feature a three-carbon spacer separating the imidazole pharmacophore and the aromatic 1,3-benzodioxole pharmacophore part. The assigned chemical structures of the target compounds **5a**–**o** were assured with the aid of various spectroscopic approaches. In addition, single crystal X-ray analysis of compound **5e** confirmed unequivocally the assigned chemical structures of the title compounds **5a**–**o** and established the (*E*)-configuration of their imine functionality. 

## 2. Results and Discussion

### 2.1. Chemistry

**Scheme 1:**
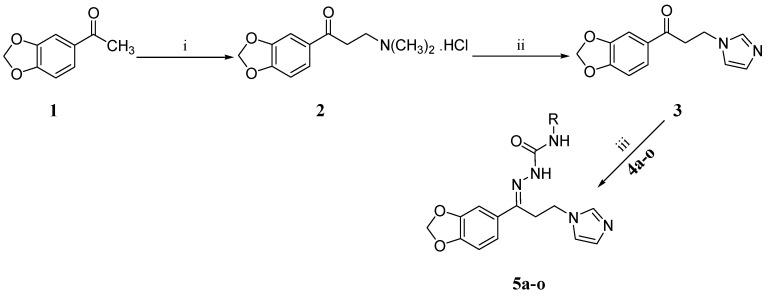
Synthesis of the target semicarbazones **5a**–**o**. *Reagents and conditions*: (i) HN(CH_3_)_2_·HCl, (CH_2_O)_n_, conc. HCl, ethanol, reflux, 2 h; (ii) Imidazole, water, reflux, 5 h; (iii) Appropriate semicarbazide **4a**–**n**, ethanol, acetic acid, rt, 18 h or semicarbazide hydrochloride (**4o**), anhydrous sodium acetate, ethanol, rt, 18 h for **5o**.

Scheme 1 presents the pathway which was adopted to prepare the target compounds **5a**–**o**. The reaction sequence commenced using the commercially available acetophenone derivative **1** to afford the ketone **3** according to the previously reported procedure [19]. The appropriate semicarbazides **4a**–**o** were added to the ketone **3** with water elimination under mild conditions to furnish the respective semicarbazones **5a**–**o** in moderate yields. The mild synthetic conditions used to obtain the target semicarbazones **5a**–**o** plays the pivotal role to get the pure geometrical (*E*)-isomers of compounds **5a**–**o** as confirmed by the single crystal X-ray analysis of compound **5e** as a representative example of this type of compounds. The assigned chemical structures of the title compounds **5a**–**o** were assured via different spectroscopic tools. Thus, they displayed IR bands in the range of 3390–3209, 1793–1645, 1664–1506 cm^−1^, corresponding to NH, C=O, and C=N, respectively. The ^1^H-NMR spectra of compounds **5a**–**o** showed triplets in the δ = 3.19–3.53 ppm range which were assigned to the two protons of the methylenes of (CH_2_–CH_2_–N) and other triplets appeared at δ = 4.02–4.30 ppm for the protons of the other methylenes (CH_2_–CH_2_–N). The benzodioxole protons (–O–CH_2_–O–) were observed as singlets in the range of δ = 6.05–6.09 (ppm). The imidazole protons (–N–CH=CH–N=) were noticed as singlet signals in the range of δ = 6.85–6.97 ppm, another singlet signals appeared in the range of δ = 7.17–7.37 ppm for the imidazole protons (–N–CH=CH–N=), while the third imidazole protons (–N–CH=N–) were noted as singlet signals in the range of δ = 7.61–7.86 ppm. The aromatic protons appeared in the region of δ = 6.61–8.25 ppm. Additionally, two singlet signals in the range of δ = 7.44–9.73 and 9.76–10.63 ppm were assigned for the two protons of the semicarbazone (NH) groups. The amine protons (NH₂) of compound **5o** were observed as a singlet at δ = 6.56 ppm.

The ^13^C-NMR spectra of the semicarbazones **5a**–**o** exhibited signals in the δ = 28.5–28.9, 42.6–43.1, 101.7–101.9, 119.9–120.8, 131.4–136.8, and 137.3–137.8 ppm range indicating (CH_2_-CH_2_-N), (CH_2_-CH_2_-N), (O–CH_2_–O–), (-N–CH=CH–N=), (–N–CH=CH–N=), and (–N–CH=N–) carbons, respectively. While the aromatic carbons appeared in the δ = 106.3–148.3 ppm range, the carbonyl (C=O) carbons were noted in the range of δ = 148.2–152.1 ppm and the (C=N) carbons were observed in the δ = 148.8–155.7 ppm range.

The mass (ESI) spectra of compounds **5a**–**o** displayed their anticipated molecular ion peaks in the protonated forms [M + 1]^+^, except for compounds **5a**, **5k**, **5m**, and **5o** which showed their anticipated molecular ion peaks in the deprotonated forms [M − 1]^−^ due to their measurement in the negative (ESI) mode. 

### 2.2. Crystal Structure of Compound ***5e***

**Figure 1:**
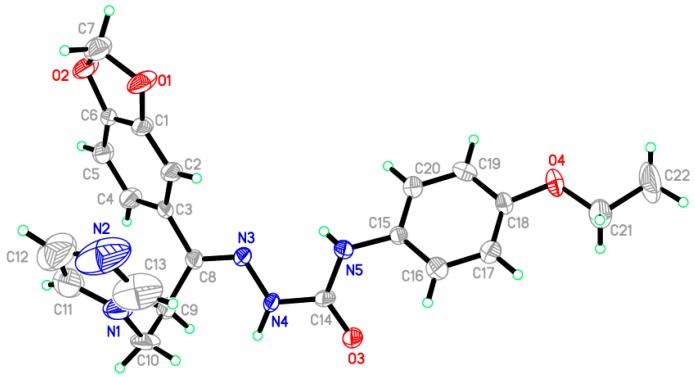
ORTEP diagram of compound **5e**. Displacement ellipsoids are plotted at the 40% probability level for non-H atoms.

The crystallographic data and refinement information of compound **5e** (C_22_H_23_N_5_O_4,_) are summarized in Table S1. The selected bond lengths and bond angles are listed in Table 1. The asymmetric unit is containing one independent molecule as shown in Figure 1. 

All the bond lengths and angles are within normal ranges [20]. Compound **5e** was found in th*e* (*E*)-configuration regarding its imine double bond C8=N3 as shown in Figure 1. The molecules are packed together in the crystal structure along the *b* axis by one classical hydrogen bond between N4-H1N1^.....^O3 and three non-classical hydrogen bonds, where C5, C9 and C21 atoms act as H-donors and O1, O3 and N2 act as H- acceptors. In addition, one intra-molecular hydrogen bond between N5-H1NB^.....^N3 which stabilizes the configuration of the molecule (Figure S1 and Table 2). The 1,3-benzodioxole plane forms dihedral angles of 64.76° and 23.43° with the ethoxyphenyl ring and imidazole ring, respectively.

### 2.3. Antifungal Activity of the Target Semicarbazones ***5a**–**o***

Table 3 presents the antifungal potential of the target semicarbazones 5**a**–**o** against the four tested fungal strains measured as the diameter of the inhibition zone (DIZ) and minimum inhibitory concentration (MIC) assay results. Compounds **5a**–**o** manifested moderate to good activity in the DIZ assay toward the four tested fungi, with DIZ values in the range of 11–23 mm. Compound **5e**, bearing a 4-ethoxyphenyl fragment, showed the best MIC value (0.304 µmol/mL) against both *C. tropicalis* and *C. parapsilosis* species. Moreover, compound **5b**, bearing a 4-bromophenyl moiety, is the most active congener among the synthesized semicarbazones **5a**–**o** toward *A. niger*, with a MIC value of 0.561 µmol/mL, followed by the equipotent dichlorinated compounds **5l** and **5m** with MIC values of 0.574 µmol/mL. Compounds **5b** and **5f** are the weakest compounds among the tested semicarbazones **5a**–**o** against both *C. albicans* and *C. tropicalis*, with MIC values of more than 1.12 and 1.30 µmol/mL, respectively, whereas, compound 5d is the weakest one against both *C.* *parapsilosis* and *A. niger* with a MIC value of more than 1.12 µmol/mL. It seems that the antifungal activity of the semicarbazones **5a**–**o** was not favorable in the cyclohexane-bearing semicarbazone, compound **5n**, or in the unsubstituted semicarbazone, compound 5o. Moreover, the *para*-substitution with halogen, methyl, or alkoxy groups (compounds **5b**, **5d**–**g** and **5j**) did not give good anti-*C. albicans* activity in the synthesized semicarbazones 5**a**–**o**, whereas, the *meta*-substitution with electron withdrawing groups (compounds **5c** and **5k**) or the dichlorinated analogue (compound **5l**) displayed the best anti-*C. albicans* activity. 

## 3. Experimental

### 3.1. General Information

Melting points were measured using Gallenkamp melting point device, and are uncorrected. A Perkin Elmer BX FT-IR spectrometer (Perkin Elmer, Shelton, CT, USA) was used to record the infrared (IR) spectra from KBr disks. A Bruker NMR spectrometer (Bruker, Reinstetten, Germany) was used to carry out the NMR measurements of the prepared samples in DMSO-*d*_6_ at 500 MHz for ^1^H and 125.76 MHz for ^13^C. Chemical shifts are indicated in δ-values (ppm) in relation to TMS used as an internal standard. Elemental analyses of the target compounds were obtained using a Vario EL III analyzer (Langenselbold, Darmstadt, Germany) and the results agreed favorably with the proposed structures within ± 0.4% of the theoretical values. Mass spectra were obtained using an Agilent Quadrupole 6120 LC/MS (Agilent Technologies, Palo Alto, CA, USA) with an ESI (electrospray ionization) source. Silica gel TLC (thin layer chromatography) fluorescent plates were secured from Merck (Darmstadt, Germany) and visualization was achieved by illumination with a UV light source (254 nm). 

### 3.2. Synthesis

#### 3.2.1. General Procedure for the Synthesis of Semicarbazones **5a**–**n**

A reaction mixture containing the appropriate semicarbazide **4a**–**n** [21,22] (10 mmol), the ketone **3** (0.24 g, 10 mmol), and few drops of glacial acetic acid in absolute ethanol (15 mL) was stirred at room temperature for 18 h. The reaction mixture was evaporated under reduced pressure and the residue was purified by re-crystallization from ethanol to furnish the corresponding semicarbazones **5a**–**n**.

*(2E)-2-[1-(1,3-Benzodioxol-5-yl)-3-(1H-imidazol-1-yl)propylidene]-N-phenylhydrazinecarboxamide* (**5a**). Yield 0.26 g (26%); white powder m.p. 162–164 °C; IR (KBr): ν (cm^−1^) 3385 (NH), 3198, 2968, 1793 (C=O), 1621 (C=N), 1521, 1262, 756; ^1^H-NMR (DMSO-*d*_6_): δ (ppm) 3.29 (s, 2H, –CH_2_–CH_2_–N), 4.14 (s, 2H, –CH_2_–CH_2_–N), 6.07 (s, 2H, –O–CH_2_–O–), 6.90 (d, *J* = 8.0 Hz, 1H, Ar–H), 6.97 (s, 1H, –N–CH=CH–N=), 7.04 (t, *J* =6.5 Hz, 1H, Ar–H), 7.27–7.33 (m, 3H, Ar–H, –N–CH=CH–N=), 7.38 (s, 1H, Ar–H), 7.61–7.63 (m, 3H, Ar–H), 7.86 (s, 1H, –N–CH=N–), 8.98 (s, 1H, NH), 10.18 (s, 1H, NH); ^13^C-NMR (DMSO-*d*_6_): *δ* (ppm) 28.6 (–CH_2_–CH_2_–N), 43.1 (–CH_2_–CH_2_–N), 101.7 (–O–CH_2_–O–), 107.1, 108.3 (Ar-CH), 120.4 (–N–CH=CH–N=), 120.6, 121.2, 123.0, 127.5, 128.9, 131.6 (Ar-CH, Ar–C, –N–CH=CH–N=), 137.6 (–N–CH=N–), 139.5, 145.1, 148.2, 148.5, 154.0 (Ar–C, C=O, C=N); MS *m*/*z* (ESI): 376.0 [M − H]^−^.

*(2E)-2-[1-(1,3-Benzodioxol-5-yl)-3-(1H-imidazol-1-yl)propylidene]-N-(4-bromophenyl)hydrazine carboxamide* (**5b**). Yield 0.37 g (37%); white powder m.p. 193–195 °C; IR (KBr): ν (cm^−1^) 3342 (NH), 3211, 2968, 1751 (C=O), 1664 (C=N), 1442, 1251, 755; ^1^H-NMR (DMSO-*d*_6_): δ (ppm) 3.28 (t, *J* = 7.0 Hz, 2H, –CH_2_–CH_2_–N), 4.09 (t, *J* = 7.0 Hz, 2H, –CH_2_–CH_2_–N), 6.07 (s, 2H, -O-CH_2_-O-), 6.86 (s, 1H, –N–CH=CH–N=), 6.90 (d, *J* = 8.5 Hz, 1H, Ar–H), 7.26 (d, *J* = 1.5 Hz, 1H, Ar-H), 7.27 (s, 1H, –N–CH=CH–N=), 7.44 (d, *J* = 3.5 Hz, 1H, Ar–H), 7.47 (d, *J* = 8.5 Hz, 2H, Ar-H), 7.62 (s, 1H, –N–CH=N–), 7.65 (d, *J* = 8.5 Hz, 2H, Ar-H), 9.01 (s, 1H, NH), 10.20 (s, 1H, NH); ^13^C-NMR (DMSO-*d*_6_): *δ* (ppm) 28.7 (–CH_2_–CH_2_–N), 42.7 (–CH_2_–CH_2_–N), 101.7 (-O-CH_2_-O-), 107.3, 108.2 (Ar-CH), 119.9 (–N–CH=CH–N=), 120.7, 121.4, 122.6, 128.7, 131.5, 132.0 (Ar-CH, Ar–C, –N–CH=CH–N=), 137.8 (–N–CH=N–), 140.0, 145.7, 148.1, 148.5, 154.0 (Ar–C, C=O, C=N); MS *m*/*z* (ESI): 456.0 [M + H]^+^, 457.0 [(M + 1) + H]^+^, 458.0 [(M + 2) + H]^+^, 459.0 [(M + 3) + H]^+^, 478.0 [M + 23]^+^, 494.0 [M + 39]^+^.

*(2E)-2-[1-(1,3-Benzodioxol-5-yl)-3-(1H-imidazol-1-yl)propylidene]-N-(3-chlorophenyl)hydrazine carboxamide* (**5c**). Yield 0.36 g (36%); white powder m.p. 182–184 °C; IR (KBr): ν (cm^−1^) 3367 (NH), 3086, 2935, 1676 (C=O), 1525 (C=N), 1448, 1236, 750; ^1^H-NMR (DMSO-*d*_6_): δ (ppm) 3.35 (t, *J* = 7.0 Hz, 2H, –CH_2_–CH_2_–N), 4.10 (t, *J* = 7.0 Hz, 2H, –CH_2_–CH_2_–N), 6.07 (s, 2H, –O–CH_2_–O–), 6.86 (s, 1H, –N–CH=CH–N=), 6.90 (d, *J* = 8.0 Hz, 1H, Ar–H), 7.08 (d, *J* = 6.5 Hz, 1H, Ar–H), 7.27 (br.s, 2H, –N–CH=CH–N=, Ar-H), 7.33 (t, *J* = 8.0 Hz, 1H, Ar–H), 7.63 (s, 3H, Ar-H, –N–CH=N–), 7.85 (s, 1H, Ar-H), 9.05 (s, 1H, NH), 10.23 (s, 1H, NH); ^13^C-NMR (DMSO-*d*_6_): *δ* (ppm) 28.7 (–CH_2_–CH_2_–N), 42.7 (–CH_2_–CH_2_–N), 101.7 (-O-CH_2_-O-), 107.3, 108.2, 119.0 (Ar-CH), 119.9 (–N–CH=CH–N=), 121.4, 122.6, 128.7, 130.5, 131.5, 133.3 (Ar-CH, Ar–C, –N–CH=CH–N=), 137.8 (–N–CH=N–), 141.1, 145.9, 148.1, 148.5, 154.0 (Ar-C, C=O, C=N); MS *m*/*z* (ESI): 412.0 [M + H]^+^, 413.0 [(M + 1) + H]^+^, 414.1 [(M + 2) + H]^+^, 434.1 [M + 23]^+^, 450.1 [M + 39]^+^.

*(2E)-2-[1-(1,3-Benzodioxol-5-yl)-3-(1H-imidazol-1-yl)propylidene]-N-(4-chlorophenyl)hydrazine carboxamide* (**5d**). Yield 0.21 g (21%); white powder m.p. 198–200 °C; IR (KBr): ν (cm^−1^) 3344 (NH), 3209, 2904, 1676 (C=O), 1533 (C=N), 1490, 1232, 753; ^1^H-NMR (DMSO-*d*_6_): δ (ppm) 3.27 (t, *J* = 7.0 Hz, 2H, –CH_2_–CH_2_–N), 4.09 (t, *J* = 6.5 Hz, 2H, –CH_2_–CH_2_–N), 6.07 (s, 2H, –O–CH_2_–O–), 6.86 (s, 1H, –N–CH=CH–N=), 6.90 (d, *J* = 8.0 Hz, 1H, Ar–H), 7.26–7.27 (m, 2H, Ar–H, –N–CH=CH–N=), 7.36 (d, *J* = 9.0 Hz, 2H, Ar–H), 7.62 (br.s, 2H, Ar–H, –N–CH=N–), 7.70 (d, *J* = 8.5 Hz, 2H, Ar-H ), 9.01 (s, 1H, NH), 10.20 (s, 1H, NH); ^13^C-NMR (DMSO-*d*_6_): *δ* (ppm) 28.7 (–CH_2_–CH_2_–N), 42.7 (–CH_2_–CH_2_–N), 101.7 (-O-CH_2_-O-), 107.3, 108.2 (Ar-CH), 119.9 (–N–CH=CH–N=), 121.4, 122.1, 122.2, 126.7, 128.7, 131.5 (Ar-CH, Ar–C, –N–CH=CH–N=), 137.8 (–N–CH=N–), 138.5, 145.7, 148.1, 148.5, 154.1 (Ar-C, C=O, C=N); MS *m*/*z* (ESI): 412.1 [M + H]^+^, 413.0 [(M + 1) + H]^+^, 414.1 [(M + 2) + H]^+^. 

*(2E)-2-[1-(1,3-Benzodioxol-5-yl)-3-(1H-imidazol-1-yl)propylidene]-N-(4-ethoxyphenyl)hydrazine carboxamide* (**5e**). Yield 0.42 g (42%); white powder m.p. 135–140 °C; IR (KBr): ν (cm^−1^) 3367 (NH), 3086, 2889, 1676 (C=O), 1525 (C=N), 1448, 1236, 754; ^1^H-NMR (DMSO-*d*_6_): δ (ppm) 1.33 (t, *J* = 6.9 Hz, 3H, CH_2_-C*H_3_*), 3.26 (t, *J* = 6.9 Hz, 2H, –CH_2_–CH_2_–N), 4.09 (q, *J* = 6.9 Hz, 2H, CH_2_–CH_3_), 4.30 (t, *J* = 6.6 Hz, 2H, –CH_2_–CH_2_–N), 6.06 (s, 2H, -O-CH_2_-O-), 6.86 (s, 1H, –N–CH=CH–N=), 6.89–6.91 (m, 3H, Ar–H), 7.26 (d, *J* = 8.1 Hz, 1H, Ar–H), 7.28 (s, 1H, –N–CH=CH–N=), 7.49 (d, *J* = 8.9 Hz, 2H, Ar–H), 7.63–7.65 (m, 2H, Ar-H, –N–CH=N–), 8.76 (s, 1H, NH), 10.05 (s, 1H, NH); ^13^C-NMR (DMSO-*d*_6_): *δ* (ppm) 15.2 (-CH_2_-CH_3_), 28.7 (–CH_2_–CH_2_–N), 42.7 (–CH_2_–CH_2_–N), 63.6 (–CH_2_–CH_3_), 101.7 (–O–CH_2_–O–), 107.8, 108.6, 114.6 (Ar-CH), 119.9 (–N–CH=CH–N=), 121.2, 122.9, 128.7, 131.7, 132.3 (Ar–CH, Ar–C, –N–CH=CH–N=), 137.8 (–N–CH=N–), 144.9, 148.2, 148.3, 152.1, 154.8 (Ar–C, C=O, C=N); MS *m*/*z* (ESI): 422.2 [M + H]^+^.

*(2E)-2-[1-(1,3-Benzodioxol-5-yl)-3-(1H-imidazol-1-yl)propylidene]-N-(4-fluorophenyl)hydrazine carboxamide* (**5f**). Yield 0.51 g (51%); white powder m.p. 172–174 °C; IR (KBr): ν (cm^−1^) 3390 (NH), 3194, 2904, 1683 (C=O), 1517 (C=N), 1442, 1211, 750; ^1^H-NMR (DMSO-*d*_6_): δ (ppm) 3.27 (t, *J* = 6.8 Hz, 2H, –CH_2_–CH_2_–N), 4.09 (t, *J* = 6.9 Hz, 2H, –CH_2_–CH_2_–N), 6.09 (s, 2H, –O–CH_2_–O–), 6.86 (s, 1H, –N–CH=CH–N=), 6.91 (d, *J* = 8.2 Hz, 1H, Ar–H), 7.13–7.17 (m, 2H, Ar–H, –N–CH=CH–N=), 7.62–7.27 (m, 2H, Ar–H), 7.63–7.66 (m, 4H, Ar–H, –N–CH=N–), 8.95 (s, 1H, NH), 10.14 (s, 1H, NH); ^13^C-NMR (DMSO-*d_6_*): *δ* (ppm) 28.7 (–CH_2_–CH_2_–N), 42.7 (–CH_2_–CH_2_–N), 101.7 (–O–CH_2_–O–), 107.3, 108.2 (Ar–CH), 115.4 (d, *J*_C-3′, F& C-5′, F_ = 21.6 Hz, C-3′ and C-5′), 119.9 (–N–CH=CH–N=), 121.3, 128.7, 131.6 (Ar–CH, Ar–C, –N–CH=CH–N=), 122.8 (d, *J*_C-2′, F& C-6′, F_ = 7.8 Hz, C-2′ and C-6′), 135.8 (d, *J*_C-1′, F_ = 2.3 Hz, C-1′), 137.8 (–N–CH=N–), 145.3, 148.2, 148.5, 154.3 (–N–CH=N–, Ar-C, C=O, C=N), 158.4 (d, *J*_C-4′, F_ = 238.7 Hz, C-4′); MS *m*/*z* (ESI): 396.2 [M + H]^+^, 397.1 [(M + 1) + H]^+^.

*(2E)-2-[1-(1,3-Benzodioxol-5-yl)-3-(1H-imidazol-1-yl)propylidene]-N-(4-methoxyphenyl)hydrazine carboxamide* (**5g**). Yield 0.51 g (51%); white powder m.p. 161–163 °C. The spectral data of compound **5g** are consistent with previously reported data [23]. 

*(2E)-2-[1-(1,3-Benzodioxol-5-yl)-3-(1H-imidazol-1-yl)propylidene]-N-(2-methylphenyl)hydrazine carboxamide* (**5h**). Yield 0.65 g (65%); white powder m.p. 177–179 °C; IR (KBr): ν (cm^−1^) 3257 (NH), 2939, 2827, 1645 (C=O), 1562 (C=N), 1450, 1236, 751; ^1^H-NMR (DMSO-*d_6_*): δ (ppm) 2.28 (s, 3H, C*H_3_*), 3.28 (t, *J* = 7.0 Hz, 2H, –CH_2_–CH_2_–N), 4.11 (t, *J* = 7.0 Hz, 2H, –CH_2_–CH_2_–N), 6.07 (s, 2H, –O–CH_2_–O–), 6.87 (s, 1H, –N–CH=CH–N=), 6.91 (d, *J* = 8.0 Hz, 1H, Ar–H), 7.03–7.06 (m, 1H, Ar–H ), 7.20–7.26 (m, 3H, Ar–H), 7.29 (s, 1H, –N–CH=CH–N=), 7.55 (s, 1H, Ar–H), 7.64 (s, 1H, –N–CH=N–), 7.74 (d, *J* = 7.5 Hz, 1H, Ar–H), 8.63 (s, 1H, NH), 10.25 (s, 1H, NH); ^13^C-NMR (DMSO-*d_6_*): *δ* (ppm) 17.9 (–CH_3_), 28.8 (–CH_2_–CH_2_–N), 42.7 (–CH_2_–CH_2_–N), 101.8 (–O–CH_2_–O–), 106.7, 108.3 (Ar-CH), 119.9 (–N–CH=CH–N=), 121.1, 123.2, 124.2, 126.6, 128.7, 130.2, 130.6, 131.6 (Ar-CH, Ar–C, –N–CH=CH–N=), 137.3 (–N–CH=N–), 137.8, 144.8, 148.3, 148.5, 154.2 ( Ar–C, C=O, C=N); MS *m*/*z* (ESI): 392.2 [M + H]^+^.

*(2E)-2-[1-(1,3-Benzodioxol-5-yl)-3-(1H-imidazol-1-yl)propylidene]-N-(3-methylphenyl)hydrazine carboxamide* (**5i**). Yield 0.54 g (54%); white powder m.p. 176–179 °C; IR (KBr): ν (cm^−1^) 3367 (NH), 3099, 2893, 1687 (C=O), 1546 (C=N), 1433, 1236, 750; ^1^H-NMR (DMSO-*d*_6_): δ (ppm) 2.31 (s, 3H, C*H_3_*), 3.27 (t, *J* = 7.0 Hz, 2H, –CH_2_–CH_2_–N), 4.10 (t, *J* = 7.0 Hz, 2H, –CH_2_–CH_2_–N), 6.07 (s, 2H, -O-CH_2_-O-), 6.86 (s, 2H, –N–CH=CH–N=, Ar–H), 6.91 (d, *J* = 8.5 Hz, 1H, Ar–H), 7.17–7.21 (m, 1H, Ar–H), 7.25 (d, *J* = 1.5 Hz, 1H, Ar-H), 7.28 (s, 1H, –N–CH=CH–N=), 7.45 (s, 2H, Ar–H ), 7.60 (d, *J* = 1.5 Hz, 1H, Ar–H), 7.63 (s, 1H, –N–CH=N–), 8.80 (s, 1H, NH), 10.11 (s, 1H, NH); ^13^C-NMR (DMSO-*d*_6_): *δ* (ppm) 21.6 (CH_3_), 28.7 (–CH_2_–CH_2_–N), 42.7 (–CH_2_–CH_2_–N), 101.7 (–O–CH_2_–O–), 107.2, 108.3, 117.8 (Ar–CH), 119.9 (–N–CH=CH–N=), 121.1, 121.3, 123.8, 128.7, 128.8, 131.6 (Ar-CH, Ar–C, –N–CH=CH–N=), 137.8 (–N–CH=N–), 138.1, 139.3, 145.3, 148.1, 148.5, 154.0 (Ar–C, C=O, C=N); MS *m*/*z* (ESI): 392.2 [M + H]^+^.

*(2E)-2-[1-(1,3-Benzodioxol-5-yl)-3-(1H-imidazol-1-yl)propylidene]-N-(4-methylphenyl)hydrazine carboxamide* (**5j**). Yield 0.43 g (43%); white powder m.p. 149–151 °C; IR (KBr): ν (cm^−1^) 3219 (NH), 3105, 2904, 1664 (C=O), 1506 (C=N), 1446, 1307, 752; ^1^H-NMR (DMSO-*d*_6_): δ (ppm) 2.28 (s, 3H, C*H_3_*) 3.27 (t, *J* = 7.0 Hz, 2H, –CH_2_–CH_2_–N), 4.09 (t, *J* = 7.0 Hz, 2H, –CH_2_–CH_2_–N), 6.07 (s, 2H, –O–CH_2_–O–), 6.86 (s, 1H, –N–CH=CH–N=), 6.90 (d, *J* = 8.5 Hz, 1H, Ar–H), 7.12 (d, *J* = 8.0 Hz, 2H, Ar–H), 7.26 (d, *J* = 8.5 Hz, 1H, Ar–H), 7.28 (s, 1H, –N–CH=CH–N=), 7.51 (d, *J* = 8.0 Hz, 2H, Ar–H), 7.61 (s, 1H, Ar-H), 7.63 ( s, 1H, –N–CH=N–), 8.79 (s, 1H, NH), 10.07 (s, 1H, NH); ^13^C-NMR (DMSO-*d*_6_): *δ* (ppm) 20.9 (CH_3_), 28.7 (–CH_2_–CH_2_–N), 42.7 (–CH_2_–CH_2_–N), 101.7 (-O-CH_2_-O-), 107.2, 108.3 (Ar-CH), 119.9 (–N–CH=CH–N=), 120.8, 121.2, 128.7, 129.3, 131.6, 131.9, 136.8 (Ar-CH, Ar–C, –N–CH=CH–N=), 137.8 (–N–CH=N–), 145.1, 148.2, 148.4, 154.1 (Ar-C, C=O, C=N); MS *m*/*z* (ESI): 392.2 [M + H]^+^, 414.1 [M + 23]^+^, 430.1 [M + 39]^+^.

*(2E)-2-[1-(1,3-Benzodioxol-5-yl)-3-(1H-imidazol-1-yl)propylidene]-N-(3-(trifluoromethyl)phenyl) hydrazinecarboxamide* (**5k**). Yield 0.51 g (51%); white powder m.p. 192–194 °C; IR (KBr): ν (cm^−1^) 3209 (NH), 3088, 2904, 1670 (C=O), 1543 (C=N), 1448, 1236, 750; ^1^H-NMR (DMSO-*d*_6_): δ (ppm) 3.31 (t, *J* = 6.6 Hz, 2H, –CH_2_–CH_2_–N), 4.13 (t, *J* = 6.5 Hz, 2H, –CH_2_–CH_2_–N), 6.07 (s, 2H, -O-CH_2_-O-), 6.92 (d, *J* = 8.2 Hz, 1H, Ar–H), 6.94 (s, 1H, –N–CH=CH–N=), 7.29 (dd, *J* = 1.2, 8.5 Hz, 1H, Ar–H), 7.35–7.37 (m, 2H, Ar–H, –N–CH=CH–N=), 7.53–7.56 (m, 1H, Ar–H), 7.64 (s, 1H, –N–CH=N–), 7.80 (s, 1H, Ar-H), 7.96 (d, *J* = 7.4 Hz, 1H, Ar–H), 8.12 (s, 1H, Ar-H), 9.30 (s, 1H, NH), 10.31 (s, 1H, NH); ^13^C-NMR (DMSO-*d*_6_): *δ* (ppm) 28.7 (–CH_2_–CH_2_–N), 43.0 (–CH_2_–CH_2_–N), 101.8 (-O-CH_2_-O-), 107.3, 108.3 (Ar-CH), 116.5, 119.2 120.3, 121.4, 123.7, 124.1, 125.8, 127.8, 129.9, 131.5 (Ar-CH, Ar–C, –N–CH=CH–N=, –N–CH=CH–N=), 137.7 (–N–CH=N–), 140.5, 145.9, 148.1, 148.6, 154.1 (Ar-C, C=O, C=N); MS *m*/*z* (ESI): 444.0 [M − H]−, 445.0 [(M + 1) − H]^−^.

*(2E)-2-[1-(1,3-Benzodioxol-5-yl)-3-(1H-imidazol-1-yl)propylidene]-N-(2,4-dichlorophenyl)hydrazine carboxamide* (**5l**). Yield 0.52 g (52%); white powder m.p. 215–217 °C; IR (KBr): ν (cm^−1^) 3338 (NH), 3089, 2910, 1691 (C=O), 1523 (C=N), 1440, 1232, 751; ^1^H-NMR (DMSO-*d*_6_): δ (ppm) 3.36 (t, *J* = 7.0 Hz, 2H, –CH_2_–CH_2_–N), 4.09 (t, *J* = 7.5 Hz, 2H, –CH_2_–CH_2_–N), 6.08 (s, 2H, –O–CH_2_–O–), 6.85 (s, 1H, –N–CH=CH–N=), 6.94 (d, *J* = 8.0 Hz, 1H, Ar–H), 7.24 (d, *J* = 8.0 Hz, 1H, Ar–H), 7.27 (s, 1H, –N–CH=CH–N=), 7.40 (s, 1H, Ar–H), 7.44 (dd, *J* = 2.0, 9.0 Hz, 1H, Ar-H), 7.62 (s, 1H, –N–CH=N–), 7.70 (d, *J* = 2.0 Hz, 1H, Ar–H), 8.24 (d, *J* = 9.0 Hz, 1H, Ar–H), 9.13 (s, 1H, NH), 10.63 (s, 1H, NH); ^13^C-NMR (DMSO-*d*_6_): *δ* (ppm) 28.9 (–CH_2_–CH_2_–N), 42.8 (–CH_2_–CH_2_–N), 101.9 (-O-CH_2_-O-), 106.3, 108.5 (Ar-CH), 119.9 (–N–CH=CH–N=), 121.2, 122.4, 123.9, 127.3, 128.4, 128.7, 129.0, 131.3, 134.9 (Ar-CH, Ar–C, –N–CH=CH–N=), 137.8 (–N–CH=N–), 146.3, 148.3, 148.8, 153.5 (Ar–C, C=O, C=N); MS *m*/*z* (ESI): 446.0 [M + H]^+^, 447.0 [(M + 1) + H]^+^, 448.1 [(M + 2) + H]^+^.

*(2E)-2-[1-(1,3-Benzodioxol-5-yl)-3-(1H-imidazol-1-yl)propylidene]-N-(3,4-dichlorophenyl)hydrazine carboxamide* (**5m**). Yield 0.37 g (37%); white powder m.p. 174–176 °C; IR (KBr): ν (cm^−1^) 3365 (NH), 3197, 3082, 1685 (C=O), 1521 (C=N), 1475, 1238, 750; ^1^H-NMR (DMSO-*d_6_*): δ (ppm) 3.30 (s, 2H, –CH_2_–CH_2_–N), 4.13 (s, 2H, –CH_2_–CH_2_–N), 6.07 (s, 2H, –O–CH_2_−O−), 6.90 (d, *J* = 8.0 Hz, 1H, Ar–H), 6.97 (s, 1H, –N–CH=CH–N=), 7.28 (dd, *J* = 1.5, 8.0 Hz, 1H, Ar–H), 7.29 (s, 1H, –N–CH=CH–N=), 7.53 (d, *J* = 9.0 Hz, 1H, Ar–H), 7.62 (s, 1H, –N–CH=N–), 7.68 (d, *J* = 7.5 Hz, 1H, Ar–H), 7.85–7.86 (m, 1H, Ar–H), 8.04 (d, *J* = 1.5 Hz, 1H, Ar–H), 9.25 (s, 1H, NH), 10.33 (s, 1H, NH); ^13^C-NMR (DMSO-*d_6_*): *δ* (ppm) 28.6 (–CH_2_–CH_2_–N), 43.1 (–CH_2_–CH_2_–N), 101.7 (–O–CH_2_–O–), 107.3, 108.2 (Ar–CH), 120.4, 120.5, 121.4, 124.3, 127.3, 127.4, 130.7, 131.1, 131.4 (Ar–CH, Ar–C, –N–CH=CH–N=, –N–CH=CH–N=), 137.6 (–N–CH=N–), 139.9, 145.9, 148.1, 148.6, 153.9 (Ar–C, C=O, C=N); MS *m*/*z* (ESI): 444.0 [M − H]^−^, 445.0 [(M + 1) − H]^−^, 446.0 [(M + 2) − H]^−^.

*(2E)-2-[1-(1,3-Benzodioxol-5-yl)-3-(1H-imidazol-1-yl)propylidene]-N-cyclohexylhydrazine carboxamide* (**5n**). Yield 0.33 g (33%); white powder m.p. 174–176 °C; IR (KBr): ν (cm^−1^) 3402 (NH), 3203, 2933, 1672 (C=O), 1527 (C=N), 1460, 1251, 752; ^1^H-NMR (DMSO-*d_6_*): δ (ppm) 1.16–1.40 (m, 5 H, cyclohexyl), 1.58–1.99 (m, 5 H, cyclohexyl), 3.19 (t, *J* = 7.0 Hz, 2H, –CH_2_–CH_2_–N), 3.56–3.57 (m, 1H, cyclohexyl), 4.02 (t, *J* = 7.0 Hz, 2H, –CH_2_–CH_2_–N), 6.05 (s, 2H, –O–CH_2_–O–), 6.61 (d, *J* = 8.0 Hz, 1H, Ar–H), 6.85 (s, 1H, –N–CH=CH–N=), 6.88 (d, *J* = 8.0 Hz, 1H, Ar–H), 7.18 (dd, *J* = 1.0, 8.0 Hz, 1H, Ar–H), 7.26 (s, 1H, –N–CH=CH–N=), 7.44 (s, 1H, NH), 7.61 (s, 1H, –N–CH=N–), 9.76 (s, 1H, NH); ^13^C-NMR (DMSO-*d*_6_): *δ* (ppm) 25.4 (–CH_2_–CH_2_–), 25.7 (–CH_2_–CH_2_–), 28.5 (–CH_2_–CH_2_–N), 33.3 (–CH_2_–CH_2_–), 42.6 (–CH_2_–CH_2_–N), 48.6 (–CH_2_–CH–CH_2_), 101.7 (-O-CH_2_-O-), 106.8, 108.3, 119.9 (Ar-CH), 120.8 (–N–CH=CH–N=), 128.7 (Ar–CH), 131.9 ( –N–CH=CH–N=), 137.8 (–N–CH=N–), 143.6, 148.1, 148.2, 155.7 (Ar–C, C=O, C=N); MS *m*/*z* (ESI): 384.2 [M + H]^+^, 406.2 [M + 23]^+^, 422.2 [M + 39]^+^.

#### 3.2.2. Synthesis of (2*E*)-2-[1-(1,3-benzodioxol-5-yl)-3-(1*H*-imidazol-1-yl)propylidene]hydrazine Carboxamide (**5o**)

A reaction mixture containing the ketone **3** (0.49 g, 2.0 mmol), semicarbazide hydrochloride (**4o**, 0.22 g, 2.0 mmol) and anhydrous sodium acetate (0.16 g, 2.0 mmol) in absolute ethanol (15 mL) was stirred at ambient temperature for 18 hrs. The reaction mixture was filtered and the filtrate was evaporated under vacuum. The residue was crystallized from ethanol to give 0.5 g (50%) of the semicarbazone **5o** as a white solid m.p. 177–179 °C. IR (KBr): ν (cm^−1^) 3442 (NH₂), 3215 (NH), 3105, 2914, 1695 (C=O), 1593 (C=N) 1499, 1269, 757; ^1^H-NMR (DMSO-*d*_6_): δ (ppm) 3.53 (t, *J* = 6.5 Hz, 2H, –CH_2_–CH_2_–N), 4.06 (t, *J* = 7.0 Hz, 2H, –CH_2_–CH_2_–N), 6.04 (s, 2H, –O–CH_2_–O–), 6.56 (br.s, 2H, NH₂), 6.86 (d, *J* = 8.0 Hz, 1H, Ar–H), 6.92 (s, 1H, –N–CH=CH–N=), 7.20 (dd, *J* = 1.5, 8.0 Hz, 1H, Ar–H), 7.32 (s, 1H, –N–CH=CH–N=), 7.54 (d, *J* = 1.5 Hz, 1H, Ar-H), 7.75 (s, 1H, –N–CH=N–), 9.73 (s, 1H, NH); ^13^C-NMR (DMSO-*d*_6_): *δ* (ppm) 28.2 (–CH_2_–CH_2_–N), 42.8 (–CH_2_–CH_2_–N), 101.6 (–O–CH_2_–O–), 106.8, 108.2 (Ar–CH), 120.2 (–N–CH=CH–N=),120.8, 127.9, 131.8 (Ar-CH, Ar–C, –N–CH=CH–N=), 137.6 (–N–CH=N–), 143.2, 148.1, 148.2, 157.8 ( Ar–C, C=O, C=N); MS *m*/*z* (ESI): 300.7 [M − H]^−^.

### 3.3. Crystal Structure Determination of Compound ***5e***

Compound **5e** was obtained as single crystals by slow evaporation from the ethanolic solution of the pure compound at room temperature. Data were collected on a Bruker APEX-II D8 Venture area diffractometer, equipped with graphite monochromatic Cu *K*α radiation, λ = 1.542 Å at 293 (2) K. Cell refinement and data reduction were carried out by Bruker SAINT. SHELXT [24] was used to solve structure. The final refinement was carried out by full-matrix least-squares techniques with anisotropic thermal data for non-hydrogen atoms on *F*. CCDC 1879726 contains the supplementary crystallographic data for this compound can be obtained free of charge from the Cambridge Crystallographic Data Centre *via* www.ccdc.cam.ac.uk/data_request/cif.

### 3.4. Antifungal Activity of the Title Semicarbazones ***5a**–**o***

The title compounds **5a**–**o** were evaluated for their *in vitro* antifungal activity using diameter of the inhibition zone (DIZ) and minimum inhibitory concentration (MIC) assays against *Candida albicans*, *Candida tropicalis* and *Aspergillus niger* according to the literature protocols [25].

## 4. Conclusions

The synthesis of certain new imidazole-semicarbazone conjugates **5a**–**o** bearing benzodioxole moieties is reported. The assigned chemical structures of the title semicarbazones **5a**–**o** have been verified with the aid of several spectroscopic approaches. The X-ray crystal structure of compound **5e** confirmed the designated chemical structure of the target compounds **5a**–**o** and confirmed the (*E*)-configuration of their imine fragments. The antifungal potential of the conjugates **5a**–**o** was assessed against four fungal strains with the aid of DIZ and MIC assays. It seems that the antifungal activity was not favored in the cyclohexane-bearing semicarbazone, compound **5n**, or in the unsubstituted semicarbazone, compound **5o**. The *meta*-substitution (compounds **5c** and **5k**) with electron withdrawing groups or the dichlorinated analogue (compound **5l**) showed the best anti-*C. albicans* activity. Our efforts are continued aiming to get new potent, broad spectrum and safe azole-bearing antifungal drug-like candidates.

## Figures and Tables

**Table:** 

**Compound No.**	**R**	**Compound No.**	**R**	**Compound No.**	**R**	**Compound No.**	**R**
**5a**	C_6_H_5_	**5e**	4-OC_2_H_5_-C_6_H_4_	**5i**	3-CH_3_-C_6_H_4_	**5m**	3,4-Cl_2_-C_6_H_3_
**5b**	4-Br-C_6_H_4_	**5f**	4-F-C_6_H_4_	**5j**	4-CH_3_-C_6_H_4_	**5n**	C_6_H_9_
**5c**	3-Cl-C_6_H_4_	**5g**	4-OCH_3_-C_6_H_4_	**5k**	3-CF_3_-C_6_H_4_	**5o**	H
**5d**	4-Cl-C_6_H_4_	**5h**	2-CH_3_-C_6_H_4_	**5l**	2,4-Cl_2_-C_6_H_3_		

**Table 1:** Selected geometric parameters (Å, °) of compound **5e.**

O1—C1	1.362 (7)	N1—C13	1.366 (14)
O1—C7	1.425 (7)	N2—C12	1.399 (18)
O2—C6	1.376 (7)	N2—C13	1.293 (17)
O2—C7	1.427 (6)	N3—N4	1.380 (7)
O3—C14	1.229 (8)	N3—C8	1.294 (7)
O4—C18	1.372 (7)	N4—C14	1.372 (8)
O4—C21	1.430 (7)	N5—C14	1.345 (9)
N1—C10	1.443 (11)	N5—C15	1.431 (8)
N1—C11	1.366 (14)		
C1—O1—C7	105.0 (4)	N3—C8—C3	115.0 (4)
C6—O2—C7	105.1 (4)	N3—C8—C9	125.8 (5)
C18—O4—C21	118.5 (4)	N1—C10—C9	112.8 (5)
C10—N1—C11	126.2 (8)	N1—C11—C12	107.5 (10)
C10—N1—C13	128.4 (8)	N2—C12—C11	109.4 (12)
C11—N1—C13	105.2 (9)	N1—C13—N2	113.3 (11)
C12—N2—C13	104.5 (11)	N4—C14—N5	116.4 (6)
N4—N3—C8	118.3 (4)	O3—C14—N4	119.5 (6)
N3—N4—C14	117.6 (5)	O3—C14—N5	124.1 (6)
C14—N5—C15	125.7 (6)	N5—C15—C20	117.9 (5)
O1—C1—C6	110.4 (5)	N5—C15—C16	122.4 (6)
O1—C1—C2	128.1 (4)	O4—C18—C17	126.2 (5)
O2—C6—C5	129.5 (4)	O4—C18—C19	114.6 (5)
O2—C6—C1	109.0 (4)	O4—C21—C22	106.5 (5)
O1—C7—O2	107.8 (5)		

**Table 2:** The geometry of hydrogen-bonding (Å, °) of compound **5e**.

*D*—H···*A*	*D*—H	H···*A*	*D*···*A*	*D*—H···*A*
N4—H1NA···O3 ^i^	1.01(6)	1.87(6)	2.857(6)	167(5)
N5—H1NB···N3	0.68(7)	2.21(7)	2.611(7)	120(7)
C5—H5A···O1 ^ii^	0.9300	2.5500	3.454(6)	164.00
C9—H9A···O3 ^i^	0.9700	2.4200	3.182(7)	135.00
C21—H21A···N2 ^iii^	0.9700	2.5700	3.500(13)	160.00

Symmetry codes: (i) −x, −y + 2, −z; (ii) x − 1, y, z; (iii) x + 1, y − 1, z.

**Table 3:** Antifungal potential of the target semicarbazones **5a**–**o** against *C. albicans*, *C. tropicalis*, *C. parapsilosis*, and *A. niger.*

Compound No.	*Candida albicans*		*Candida tropicalis*		*Candida parapsilosis*		*Asperagillus niger*	
	DIZ ± SD * (mm)	MIC (µmol/mL)	DIZ ± SD * (mm)	MIC (µmol/mL)	DIZ ± SD * (mm)	MIC (µmol/mL)	DIZ ± SD * (mm)	MIC (µmol/mL)
**5a**	14 ± 0.60	0.678	20 ± 0.90	0.339	11 ± 0.80	0.678	15 ± 0.50	0.678
**5b**	12 ± 0.70	> 1.12	18 ± 0.50	> 1.12	15 ± 0.58	>1.12	14 ± 0.40	0.561
**5c**	14 ± 0.58	0.311	20 ± 0.90	0.311	16 ± 1.10	0.622	15 ± 0.50	0.622
**5d**	14 ± 0.60	0.622	17 ± 0.50	> 1.24	14 ± 0.40	>1.24	0.0 ± 0.0	>1.24
**5e**	13 ± 0.40	0.607	22 ± 0.80	0.304	16 ± 0.12	0.304	13 ± 1.00	0.607
**5f**	14 ± 0.60	0.648	17 ± 1.00	> 1.30	14 ± 0.60	0.648	16 ± 1.10	0.648
**5g**	14 ± 0.58	0.628	22 ± 0.50	0.314	12 ± 1.20	0.628	15 ± 0.50	0.628
**5h**	14 ± 1.00	0.654	19 ± 0.50	0.654	18 ± 0.90	0.327	14 ± 1.00	0.654
**5i**	13 ± 0.40	0.654	21 ± 1.00	0.327	17 ± 0.58	0.654	13 ± 0.40	0.654
**5j**	13 ± 1.00	0.654	22 ± 1.00	0.327	17 ± 0.90	0.327	15 ± 0.58	0.654
**5k**	12 ± 0.43	0.287	23 ± 0.50	0.287	15 ± 0.60	0.575	14 ± 0.80	0.575
**5l**	14 ± 0.58	0.287	21 ± 1.00	0.574	14 ± 0.60	0.574	13 ± 0.40	0.574
**5m**	11 ± 0.30	0.574	17 ± 0.80	>1.15	16 ± 0.58	>1.15	16 ± 0.80	0.574
**5n**	14 ± 0.40	0.668	19 ± 1.00	1.34	19 ± 0.50	>1.24	13 ± 1.00	0.668
**5o**	14 ± 0.58	0.850	20 ± 0.90	0.850	15 ± 0.90	0.850	14 ± 0.10	0.850
Fluconazole	18 ± 1.10	0.051	19 ± 1.00	0.045	19 ± 0.90	0.047	ND	ND
Ketoconazole	ND	ND	ND	ND	ND	ND	29 ± 0.60	0.02

* The arithmetic mean of the inhibition zone diameters in mean ± standard deviation; ND: not determined.

## Supplementary Materials

The following are available online, Figure S1: Molecular packing of compound **5e** viewed hydrogen bonds which are drawn as dashed lines along *b* axis, Table S1: The refinement information and crystallographic data of the semicarbazone **5e**.
