# Supplementary material for: Synthesis and Spectroscopic Identification of Certain Imidazole-Semicarbazone Conjugates Bearing Benzodioxole Moieties: New Antifungal Agents
Source: Molecules. 2019 Jan 7;24(1):200. doi: 10.3390/molecules24010200 (PMC6337076; doi:10.3390/molecules24010200)
Supplement: Supplementary file 1 [file molecules-24-00200-s001.pdf]

Synthesis and spectroscopic identification of certain imidazole-semicarbazone conjugates bearing benzodioxole moiety: New antifungal agents

Reem I. Al-Wabli[1]*, Alwah R. Al-Ghamdi[1], Hazem A. Ghabbour[2], Mohamed H. Al-Agamy [3,4] and Mohamed I. Attia[1,5]*

[1]Department of Pharmaceutical Chemistry, College of Pharmacy, King Saud University, P.O. Box 2457, Riyadh 11451, Saudi Arabia
[2]Department of Medicinal Chemistry, Faculty of Pharmacy, Mansoura University, Mansoura 35516, Egypt
[3]Department of Pharmaceutics, College of Pharmacy, King Saud University, P.O. Box 2457,
Riyadh 11451, Saudi Arabia
[4]Microbiology and Immunology Department, Faculty of Pharmacy, Al-Azhar University,
Cairo 11884, Egypt
[5]Medicinal and Pharmaceutical Chemistry Department, Pharmaceutical and Drug Industries Research Division, National Research Centre (ID: 60014618), El Bohooth Street, Dokki, Giza 12622, Egypt

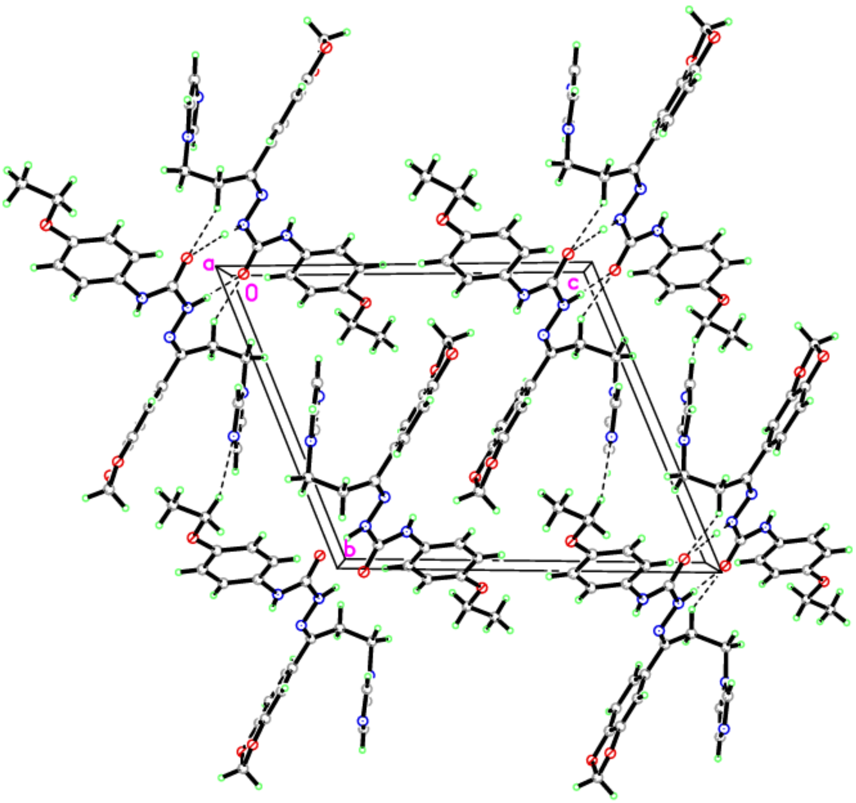

Figure S1: Molecular packing of compound **5e** viewed hydrogen bonds which are drawn as dashed lines along *b* axis.

Table S1: The refinement information and crystallographic data of the semicarbazone **5e**.

**Crystal data**	
Molecular formula	$C_{22}H_{23}N_5O_4$
*M*r	421.45
Crystal system, space group	Triclinic, *P-1*
Temperature (K)	293
a, *b*, *c* (Å)	6.3561 (3), 12.5095 (8), 14.5411 (9)
α, *β*, γ (°)	67.073 (4), 79.989 (4), 84.370 (4)
V (Å^3)	1048.05 (11)
Z	2
Radiation type	Cu *K*α
μ (mm^{-1})	0.78
Crystal size (mm)	0.42 × 0.13 × 0.06
**Data collection**	
Diffractometer	Bruker APEX-II D8 venture diffractometer
Absorption correction	Multi-scan SADABS Bruker 2014

T_{min}, T_{max}	0.897, 0.923
No. of measured, independent and observed [I> 2σ(I)] reflections	4803, 2189, 1142
R_{int}	0.051
**Refinement**	
R[F^2> 2σ(F^2)], wR(F^2), S	0.062, 0.165, 1.01
No. of reflections	2189
No. of parameters	290
No. of restraints	0
H-atom treatment	H atoms treated by a mixture of independent and constrained refinement
$\Delta\rho_{max}$, $\Delta\rho_{min}$ (e Å^{-3})	0.16, −0.17

Antifungal Activity

Materials

The reference standard antifungal drug, ketoconazole, was purchased from Sigma-Aldrich Co. (St. Louis, MO, USA). Liquid RPMI 1640 medium supplemented with L-glutamine was obtained from Gibco-BRL, Life Technologies (Paisley, Scotland). Sabouraud Dextrose Agar (SDA) was obtained from Merck Co. (Darmstadt, Germany). Dimethyl sulfoxide (100%) was used to dissolve ketoconazole, and/or the tested compound 4 to give an initial concentration of 2048 mg/L.

Organisms

The used fungal strains are *Candida albicans* (ATCC 90028), *Candida tropicalis* (ATCC 66029), *Candida parapsilosis* (ATCC 22019) and *Aspergillus niger* (ATCC 16404).

Preparation of Fungal Inocula

The inocula of the standard mold *Aspergillus niger* strain have been prepared by removing the sporulated *A. niger* from the Sabouraud Dextrose agar slant with a microbiological loop and the spores have been suspended in 10 mL of sterile water. The suspension has been filtered through sterile gauze to remove hyphae. The resulting suspension of conidia has been vigorously mixed using a vortex. The suspension has been adjusted to 1 × 105 CFU/mL using spectrophotometer. This fungal suspension has been diluted 1:5 with RPMI medium to obtain suspensions having 2× of the required final concentration. This conidial suspension had a final concentration of 1 × 104 CFU/mL when mixed with the tested solution of compound 4. On the other hand, the inocula of the standard yeast strains of *C. albicans*, *C.*

tropicalis and *C. parapsilosis* have been prepared by suspending five representative colonies, obtained from 24 to 48 h culture on Sabauraud Dextrose agar medium, in sterile distilled water. The final inoculum concentration must be between 0.5 × 105 and 2.5 × 105 CFU/mL.

Preparation of the Tested Compound Solution

Briefly, a twofold dilution series of the tested compounds has been prepared in a double strength RPMI 1640 culture medium. Ten serial dilutions were prepared to give concentrations ranged from 1024 mg/L to 2 mg/L.

Antifungal Susceptibility Studies

Minimum Inhibitory Concentrations (MICs) have been determined by broth microdilution testing as described previously by EUCAST [1]. The experiment was carried out in duplicate. Briefly, one mL of RPMI 1640 medium from each of the bottle containing the corresponding concentration of the tested compounds has been transferred into sterile 7 mL Sterilin tubes (Thermo Fisher Scientific, Waltham, MA, USA). The RPMI 1640 medium containing 1024 mg/L of the tested compounds has been dispensed to tube 1, the medium containing 512 mg/L has been dispensed to tube 2, the medium containing 256 mg/L has been dispensed to tube 3 and so on to tube 10 for the medium containing 2 mg/L of the tested compounds. One mL of the medium has been dispensed in tubes 11 (positive control) and 12 (negative control). One mL of the diluted inoculum suspension has transferred to each tube except tube 12 to bring the tested compounds dilutions to the required final test concentrations. The tubes were incubated at 35 ˚C for 72 h. The MICs of the tested compounds were determined visually by recording the degree of growth inhibition in each tube**References**

1. Rodriguez-Tudela, J.L.; Arendrup, M.C.; Barchiesi, F.; Bille, J.; Chryssanthou, E.; Cuenca-Estrella, M.; Dannaoui, E.; Denning, D.W.; Donnelly, J.P.; Dromer, F.; et al. EUCAST Definitive Document EDef 7.1: method for the determination of broth dilution MICs of antifungal agents for fermentative yeasts: Subcommittee on Antifungal Susceptibility Testing (AFST) of the ESCMID European Committee for Antimicrobial Susceptibility Testing (EUCAST). Clin. Microbiol. Infect. 2008, 14, 398–405.
